# The Treatment of Neurasthenics

**Published:** 1918-05-18

**Authors:** 


### THE TREATMENT OF NEURASTHENICS.

THE proposed change in the use of the Golders Green Home of Recovery for Shell-Shock Discharged Sailors and Soldiers is another instance of the waste of war. This institution was specially prepared for neurasthenic patients. As is usual in such an institution, the sleeping accommodation is all on the first floor, and the ground floor is devoted to recreation. From an announcement made some time ago by Mr. Hodge the Home is to be used for another class of case; therefore the living arrangements, which are suited to no other group of patients, will have to be reversed, presumably at considerable expense. The reason for the change, as announced by the Minister of Pensions in the House of Commons, is that the patients must be removed from the danger of air raids. There appears to be no question as to the success of the Home of Recovery under the management of the Hospital for Paralysis and Epilepsy; the reports, said Mr. Hodge, are satisfactory. But the medical advisers of the Ministry, who appear to differ from other medical men of experience, are firm on the point.

The whole question was raised in the House of Lords on Tuesday night by Lord Knutsford, who asked why it was proposed to close the Home of Recovery for Neurasthenic Pensioners at Golders Green. He said that "the proposal had come as a bombshell to the medical profession and all interested in the cure of neurasthenics. The hospital was one of the most, if not the most, successful in the country; £11,000 had been spent upon it by the Red Cross Society, who had no notice from the Pensions Minister that it was to be closed. In the last seven months, out of 189 patients 183 had been returned fit to work, and were in work to-day. One would think that a hospital with such a record would be encouraged; on the contrary, it was to be scrapped. One explanation was that it was in the air-raid area; yet it was to be used for men who had become paralysed in consequence of injuries in the war. As a matter of fact, men suffering from shell-shock were not distressed by air-raids, and only a small minority were disturbed by the noise of the guns. It was helpful to sit by these men and explain that there was no reason to fear, and so assist them to regain their courage. If there was one class of person who ought not to be in an air-raid area it was that affected by paralysis who could not move in time of danger. Another reason given by the Minister of Pensions for the closing of the Home was that he was going to start an institution in the country, some distance from London. There were a limited number of medical men who had studied neurasthenic conditions; they could be almost counted on the fingers of the two hands. It was quite impossible for these doctors to attend patients at a distance. Sometimes they had to sit by the men for half an hour or an hour before they could have any effect on them. It was quite impossible for ordinary doctors to attend neurasthenic patients, because they had not the experience of those who had made the affection a special study. It was said that 50 per cent. of the pensioners who had been asked had refused to go to Golders Green. A great deal depended upon how it was put to a man. If he were asked, "Would you like to go where there is likely to be an air raid, or to a Home by the beautiful River Thames?" they could imagine the answer. These men had been treated and misunderstood by doctors who had never studied the disease, and had been thought to be malingerers." The Earl of Crawford (Lord Privy Seal) replied that the Minister of Pensions had resolved upon the change on the recommendation of his medical advisers. A hospital for the treatment of shell-shock within the sound of anti-aircraft guns was undesirable. Accommodation had been procured near Maidenhead. It was true that a large number of men refused to enter the Golders Green Home because it was in the air-raid area, and as they were discharged soldiers they were free to exercise their will. This class of cases had largely diminished owing to the treatment adopted by the military authorities in France, and would be still further diminished.

